# Evolutionary analyses of KCNQ1 and HERG voltage-gated potassium channel sequences reveal location-specific susceptibility and augmented chemical severities of arrhythmogenic mutations

**DOI:** 10.1186/1471-2148-8-188

**Published:** 2008-06-30

**Authors:** Heather A Jackson, Eric A Accili

**Affiliations:** 1 Department of Cellular and Physiological Sciences, Faculty of Medicine, University of British Columbia, Vancouver, BC, V6T 1Z3, Canada

## Abstract

**Background:**

Mutations in HERG and KCNQ1 potassium channels have been associated with Long QT syndrome and atrial fibrillation, and more recently with sudden infant death syndrome and sudden unexplained death. In other proteins, disease-associated amino acid mutations have been analyzed according to the chemical severity of the changes and the locations of the altered amino acids according to their conservation over metazoan evolution. Here, we present the first such analysis of arrhythmia-associated mutations (AAMs) in the HERG and KCNQ1 potassium channels.

**Results:**

Using evolutionary analyses, AAMs in HERG and KCNQ1 were preferentially found at evolutionarily conserved sites and unevenly distributed among functionally conserved domains. Non-synonymous single nucleotide polymorphisms (nsSNPs) are under-represented at evolutionarily conserved sites in HERG, but distribute randomly in KCNQ1. AAMs are chemically more severe, according to Grantham's Scale, than changes observed in evolution and their severity correlates with the expected chemical severity of the involved codon. Expected chemical severity of a given amino acid also correlates with its relative contribution to arrhythmias. At evolutionarily variable sites, the chemical severity of the changes is also correlated with the expected chemical severity of the involved codon.

**Conclusion:**

Unlike nsSNPs, AAMs preferentially locate to evolutionarily conserved, and functionally important, sites and regions within HERG and KCNQ1, and are chemically more severe than changes which occur in evolution. Expected chemical severity may contribute to the overrepresentation of certain residues in AAMs, as well as to evolutionary change.

## Background

Two voltage-gated potassium ion channel genes, KCNQ1 and KCNH2 (HERG), encode for channels that underlie the slowly- and rapidly-activated delayed rectifier potassium currents (I_Ks _and I_Kr_), respectively [1-4]. Efflux of potassium ions through these channels is critical for repolarization of the cardiac action potential. Mutations that disrupt normal biosynthesis and function of KCNQ1 and HERG have been associated with three cardiac arrhythmias: Short QT syndrome (SQTS) [5-7], atrial fibrillation [8,9] and Long QT syndrome (LQTS) [10,11]. To date, there are close to 200 reported arrhythmia-associated mutations (AAMs) in each channel, with more than 95% of which are linked to LQTS. This arrhythmia, which affects an estimated 1 in 5000–10000 people worldwide, is characterized by a prolongation of the QT interval on an electrocardiogram and can lead to Torsade de Pointes (TdP), ventricular fibrillation and sudden cardiac death [12,13]. More recently, the progression from LQTS into TdP has been proposed as a cause of sudden infant death syndrome (SIDS) [14-16] and sudden unexplained death syndrome (SUDS) [17]. Ninety percent of all known LQTS-associated mutations occur in HERG and KCNQ1.

In studies of larger groups of proteins, or individual proteins other than HERG and KCNQ1, disease-associated amino acid mutations (DAMs) have been analyzed according to the chemical severity of the change, as determined from Grantham's Scale [18], and the location and/or context of the altered amino acid [19]. An up to two-fold increase in clinically observable disease occurs in parallel with increases in the amount of chemical change [20]. In many proteins, DAMs are chemically more severe than changes over the course of evolution (called *interspecific *changes) and polymorphic changes [21,22]. In rhodopsin, the chemical severity of DAMs also correlates with the expected chemical severity of a given codon [21], as determined by comparison with the normally occurring human codon.

The importance of an amino acid to protein function can be inferred from its conservation over the course of metazoan evolution. In many proteins, DAMs are overabundant at evolutionarily conserved and slowly evolving sites [21-24], presumably because sites that have experienced little interspecific variation are critical for function. Mutations at these sites are likely deleterious and would be removed from the population by natural selection, if given enough time. In some proteins, DAMs are unevenly distributed among functionally conserved domains [25], even after accounting for the length and number of evolutionarily conserved amino acids. These data imply that functionally conserved domains, like conserved sites, are less tolerant to mutations because of their greater importance to the overall protein function. However, according to the Neutral Theory of Molecular Evolution, most nucleotide substitutions are phenotypically neutral and avoid natural selection [26]. Most nucleotide changes, except for disease-associated changes, might then be expected to distribute randomly throughout a protein. In a study of a large number of proteins, synonymous single nucleotide polymorphisms (sSNPs) were shown to distribute randomly consistent with a neutral phenotype [23]. Initial studies of non-synonymous (ns) SNPs in the cystic fibrosis transmembrane regulator and tuberous sclerosis complex 2 gene, were also shown to distribute randomly [22]. However, a more recent, study, using many proteins, showed that nsSNPs preferentially locate at variable sites and sites with high evolutionary rates, and are underrepresented at sites that are evolutionarily conserved and have low evolutionary rates [23]. This discrepancy may be due to differences in the nature of the nsSNPs available e.g. ns SNPs are less disruptive when located at conserved sites in some proteins versus others, or to a difference in the nature of the conserved sites e.g. conserved sites in some proteins are less tolerant to amino acid changes.

In this study, we take advantage of the > 200 mutations reported per channel to quantitatively analyze, for the first time, the distribution of AAMs and non-synonymous SNPs within HERG and KCNQ1 and to determine the chemical severity of these changes, as compared to interspecific changes.

## Methods

### Sequence alignment

Human protein and mRNA sequences for HERG and KCNQ1 were obtained from NCBI. Other vertebrate sequences were collected from a BLASTP [27] search at ENSEMBL (default parameters) and the NCBI non-redundant database. To eliminate the inclusion of sequences from closely related paralogs, a manual reciprocal best-hit analysis was used. Sequences were aligned by ClustalX [28] using default settings. Less conserved regions in the N and/or C-termini were removed, leaving sequences corresponding to residues 1–906 and 83–599 of human HERG and human KCNQ1, respectively.

### Collection of arrhythmia-associated mutations (AAMs) and non-synonymous polymorphisms (nsSNPs)

HERG and KCNQ1 AAMs were obtained from the NCBI OMIM database, the Human Gene Mutation Database (HGMD), the European Society of Cardiology Working Group on Arrhythmias (WGA) LQTS gene database [29] and from primary literature [15,17,30-32]. Only missense mutations that fell within the aligned region were used, and each was included only once, to eliminate frequency bias, but multiple disease mutations could be associated with a single site. nsSNPs were collected from NCBI dbSNP, HAPMAP international and primary literature and were assumed to be phenotypically neutral based on their inclusion in the different databases. nsSNPs that overlapped with identified disease mutations were removed. The final data set included 172 AAMs and 16 nsSNPs for HERG, and 174 AAMs (Table 1) and 12 nsSNPs for KCNQ1 (see Additional file 1).

**Table 1:** Breakdown of clinical phenotype of disease mutations included in analyses

	HERG	KCNQ1
Long QT	166	169
Short QT	1	1
Atrial Fibrillation	0	1
SIDS	3	2
Long QT/SIDS	2	1

Total	172	174

### Phylogenetic analysis and determination of interspecific variability

Aligned protein sequences, with gaps removed, were used to construct a phylogenetic tree using the maximum likelihood analysis in PHYLIP [33]. SEQBOOT, PROML, and CONSENSE programs were used with a JTT model of evolution. Ancestral sequences were determined using the maximum likelihood method from PAMLv3.15 [34] under a Poisson model of amino acid evolution. Discrete values for interspecific variability (0 through n) were determined for each residue in the protein from differences between the ancestral and descendent sequences throughout the provided tree.

### Association between AAMs or nsSNPs and evolutionarily conserved sites

To determine whether AAMs and nsSNPs are preferentially associated with evolutionarily conserved sites, we compared the number of observed mutations in HERG and KCNQ1 to the number of mutations expected at each site in both proteins based on neutral substitution [22]. Sites were binned according to counts of interspecific variability (0 through i) which were determined using PAML. The expected number of mutations was determined using the following equation

(1) D_i_^expected ^= D^total ^× N_i_/N

where D_i_^expected ^is the expected number of mutations at sites that have undergone i substitutions, D^total ^is the total number of disease mutations for each channel given by ΣD_i_^observed^, N_i _is the observed number of sites in the alignment that have undergone i substitutions and N is the total number of residues in the gene being examined. Therefore, N_i_/N is the fraction of sites in the gene that belong to a particular variability class (i), and if disease mutations distribute randomly throughout the protein, then D_i_^expected ^will be proportional to the fraction of the total sites and the total number of disease mutations observed in each gene.

To determine whether differences in the distribution pattern between observed values and those expected from neutral theory were significant, the X^2 ^statistic was calculated [22] and compared to a critical value for the given degrees of freedom (i-1) using the following equation,

(2) X^2 ^= Σ_0_^i ^(D^observed^-D^expected^)^2^/D^expected ^

### Determination of codon evolutionary rate of change

The evolutionary rates of change for codons were estimated using the maximum likelihood method implemented in the CODEML program of PAML [34,23], using a discrete gamma model (eight categories). The shape parameter was either fixed or free to vary and a likelihood ratio test was performed to evaluate model fitting. Evolutionary rates based on a Poisson model of evolution were established for every site and normalized to the maximum rate observed for each protein. Values between 0 and 1 were binned into eight different categories and used to represent eight different levels of evolutionary change. The analysis was performed on nucleotide sequence alignments of core regions, with gaps removed, of human and three closely related vertebrate orthologs, guided by protein sequence alignments. The expected number of mutations at the codons belonging to each rate category was calculated using a modification of Eq. 1 where N_i _is the number of codons belonging to i category, N is the total number of amino acid positions, and D^total ^is the total number of disease mutations for each protein used in the analysis.

### Distribution of AAMs among functionally important regions of the channels

To quantify over- or underrepresentation of AAMs in functionally conserved regions of the channels, we compared their distribution to that expected from a uniform or evolutionary hypothesis [25]. First, we tested whether AAMs were distributed uniformly across the protein. The expected number of mutations in a given region was determined using the following equation

(3) D_j_^expected ^= (R_j_/R) × D^total ^

where D_j_^expected ^is the expected number of disease mutations in a particular region, j, R_j _is the number of residues found in region j, R is the total number of residues used in the analysis (ΣR_j_) and D^total ^= ΣD_j_^observed ^or the total number of disease mutations used in the analysis.

The X^2 ^statistic was calculated and compared to a critical value for the given degrees of freedom equal to j-1, where j is the number of different regions in the channel being analyzed, using the following equation,

(4) X^2 ^= Σ_j _(D^observed^-D^expected^)^2^/D^expected ^

Second, we tested whether the distribution of AAMs in different regions was related to the distribution of evolutionarily conserved sites. If AAMs are overrepresented at conserved sites, the number of AAMs for a given region will be proportional to the number of conserved sites found within that region. The expected number of mutations per region was determined using the following equation,

(5) D_j_^expected ^= Σ_0_^i ^((a_ij_/a_i_) × D_i_^observed^)

where a_i _is the total number of sites in a protein belonging to variability class i, a_ij _is the number of sites of variability class i found within the region j, and D_i_^observed ^is the total number of disease mutations found at variability sites, i, across the entire protein.

### Chemical severity of amino acid changes

The interspecific chemical severity of a given site was determined by the average severity (according to Grantham's Scale [18]) of all ancestor-descendent amino acid differences at that site throughout the tree, as reported by PAML. Only those interspecific changes that result from a single point mutation were included and each type of amino acid change at a given site was counted once to account for common ancestry [22]. The expected chemical severity at each site in HERG and KCNQ1 was determined by computing the average severity of all non-synonymous changes produced by a single point mutation from the human reference codon [21].

### Weighted average for amino acid expected chemical severity

To examine the involvement of specific amino acid residues in disease, the proportion of AAMs at a particular amino acid was calculated as percentage of the total AAMs in both channels. To determine whether these findings were due to an overrepresentation of certain amino acids in the proteins, data was normalized for the total number of codons for a particular residue. Finally, the weighted average for amino acid expected chemical severity was calculated by the sum of the average of each of the individual expected codon chemical severities of the residue multiplied by its contribution to the total number of codons for the residue in the two channels combined.

## Results

### Channel Structure and Mutation Mapping

**Figure 1:**
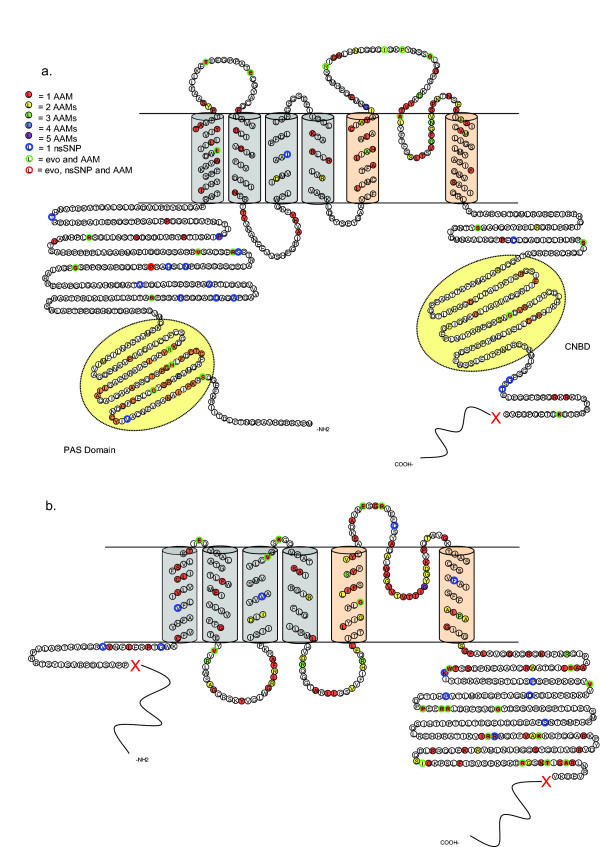
**Location of arrhythmia-associated mutations (AAMs) and non-synonymous single nucleotide polymorphisms (nsSNPs) in human HERG and KCNQ1 subunits**. a) Schematic of the human HERG subunit. Included are 172 AAMs at 134 sites and 16 nsSNPs at 16 sites. b) Schematic of KCNQ1 subunit. Included are 174 disease mutations at 130 sites and 12 nsSNPs at 12 sites. Blue region = voltage sensing domain (S1–S4), pink region = pore forming domain (S5–S6), yellow = PAS domain or CNBD domain in cytosolic regions of the channel. Red X's delimit the region used in analysis. Circles: red = 1 AAMs, yellow = 2 mutations/site, green = 3 mutations/site, blue = 4 mutations/site, purple = 5 mutations/site, blue outline = 1 nsSNP/site, green outline = overlap of disease mutation and evolutionary change, red outline = overlap of AAM, evolutionary change and nsSNP.

HERG and KCNQ1 channels are likely formed by the tetrameric assembly of individual alpha subunits, each of which is composed of six transmembrane segments and cytosolic N- and C-termini (Figure 1a, b). The voltage sensing domain (VSD) is composed of the first four transmembrane segments and a pore region is composed of S5, a re-entrant P-loop containing the selectivity filter, and S6. Mapping AAMs onto the sequences and predicted topologies of HERG and KCNQ1 subunits yielded some common distribution patterns as well as some unique to the individual proteins. The final data set used for HERG consists of 172 AAMs at 134 sites and 16 nsSNPs at 16 individual sites (see Additional file 1). Of 30 sites harboring multiple AAMs, 24 sites had two, 4 sites had three and 2 sites had four. For KCNQ1, the final data set includes 174 AAMs mapping to 130 sites and 12 nsSNPs to 12 individual sites (see Additional file 1). Thirty-six sites had multiple AAMs associated with them: 30 sites with two, 5 with three and 1 with five (Figure 1).

Between the two channels, some similarities exist in the distribution patterns of AAMs in HERG and KCNQ1, supporting results gathered when much fewer mutations were known. [35]. For example, both channels contain a large number of mutations within the pore region (23% and 29% for HERG and KCNQ1, respectively) (Table 2). On the other hand, differences in percentages of AAMs do exist in the intra- and extra-cellular linker regions as well as the two cytosolic termini. In HERG, 23% of all known disease mutations are found in the extracellular linkers (between S1 and S2, S3 and S4, S5 and the P-loop, and between the P-loop and S6). In KCNQ1, only 6% are found extracellularly whereas 20% are found in the intracellular linkers (between S2–S3 and S4–S5). These differences suggest that different linker regions contribution to overall function may be channel specific. Another difference occurs in the distal termini. In HERG, 27% of disease mutations are located in the N-terminus, compared to only 2% in KCNQ1. However, three quarters of these are located in the PAS (Per-Arnt-Sim) domain, a basic helix-loop-helix domain that is unique to the HERG N-terminus and regulates channel closing [36].

**Table 2:** Disease mutation distribution by channel region

Region	HERG	KCNQ1
N-term	6%	2%
PAS	21%	NA
VSD	9%	11%
ICL	3%	20%
ECL	23%	6%
Pore	23%	29%
C-term	15%	32%

PAS = Per-Arnt-Sim domain in HERG, N-term = N-terminal region used in analysis (excluding PAS domain in HERG), ECL = extracellular linker regions between S1–S2, S3–S4, S5-P loop and P-loop-S6, ICL = intracellular linker regions between S2–S3 and S4–S5, VSD = voltage sensing domain (only transmembrane portions), Pore = pore region including S5, P-loop and S6, C-term = C-terminal portion of channels used in this analysis.

nsSNPs distribute differently from AAMs in HERG and KCNQ1 (Figure 1). In HERG, nsSNPs are more commonly found in the cytosolic regions (15/16) compared to the transmembrane regions (1/16). In KCNQ1, 8/12 nsSNPs are found in the cytosolic regions and 4/12 nsSNPs are found in the transmembrane region. Most of these occur at sites that do not have associated disease mutations, suggesting that location is an important determinant of disease. In both channels, however, two sites situated in cytosolic regions harbor both disease and polymorphic mutations, suggesting that amino acid identity plays a role in channel dysfunction at certain sites.

### AAMs occur preferentially at sites conserved throughout vertebrate evolution and at those with lower evolutionary rates of change

The majority of AAMs mapped to sites completely conserved throughout the evolution of the respective channel: 146/172 mutations (84.9%) in HERG, mapping to 114/906 (12.6%) of the sites used in the analysis, and 153/174 (87.9%) in KCNQ1, mapping to 110/517 (21.3%) of the sites used in the analysis. Conversely, 22/172 AAMs in HERG and 20/174 AAMs in KCNQ1, map to sites with interspecific variation (Figure 1). Only two sites in HERG and one in KCNQ1 involve an amino acid change that is observed in both the AAM and in interspecific change. Because the amino acid sequences of HERG and KCNQ1 are highly conserved (over 65% complete identity between fish and human sequences in both channels for the regions used), a neutral mutational process would still produce a large number of mutations at conserved sites.

**Figure 2:**
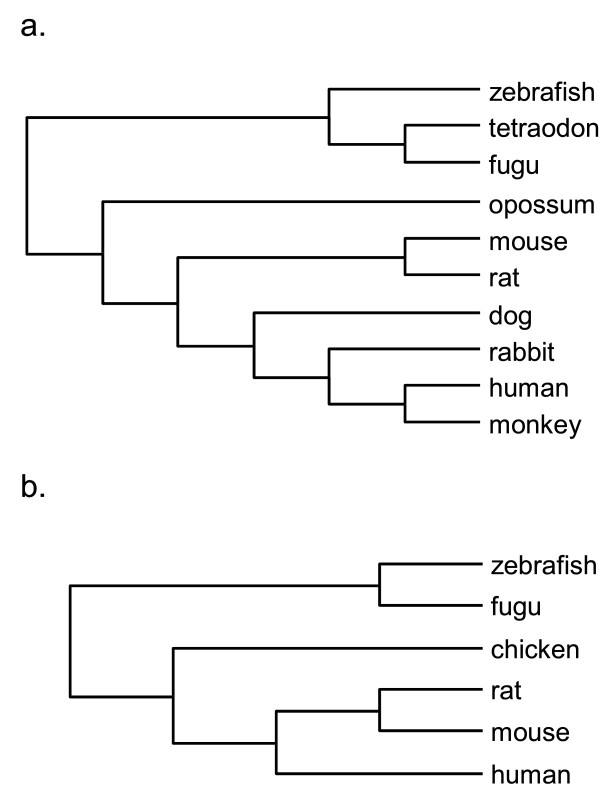
**Cladograms of vertebrate HERG and KCNQ1 protein sequences**. a) HERG: GenBank accession numbers/Ensembl identifiers for protein sequences used: human (NP_000229.1), dog (NP_001003145.1), zebrafish (NP_998002), monkey (ENSMMUP00000026900), rabbit (Q8WNY2), mouse (NP_038597.1), rat (NP_446401.1), opossum (ENSMODP00000004651), fugu (NEWSINFRUP00000161615), tetraodon (GSTENP00027811001). b) KCNQ1: GenBank accession numbers/Ensembl identifiers for KCNQ1 protein sequences used: human (NP_000209.2), mouse (NP_032460.2), rat (NP_114462.1), chicken (ENSGALP00000010441), fugu (NEWSINFRUP00000143259), zebrafish (ENSDARP00000077951). See methods for details.

To determine whether AAMs and nsSNPs occur preferentially at evolutionarily diverse sites in HERG and KCNQ1, we utilized a quantitative approach developed previously [23]. The evolutionary relationships and the number of interspecific changes at each site were determined (Figure 2) and in both channels, the largest number of interspecific changes observed at any site was five. Therefore variability data was binned into six categories, ranging from completely conserved sites (0) to highly variable (5).

**Figure 3:**
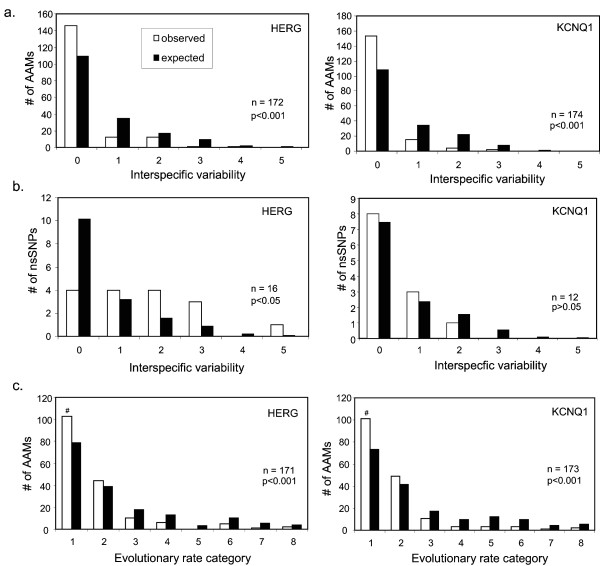
**Arrhythmia-associated mutations in HERG and KCNQ1 are overrepresented at evolutionarily conserved and slowly evolving sites**. a) Counts of observed (white bars) and expected (black bars) numbers of AAMs at amino acid sites in HERG and KCNQ1 which have undergone different numbers of substitutions among species (see methods). Because of a low number of expected mutations in the more variable positions, the X^2 ^statistic was also calculated for pooled data with two bins, 0 and 1+, with 1 degree of freedom. The number of disease mutations observed at completely conserved sites (0-class) in both HERG and KCNQ1 is significantly higher than by chance alone: HERG, X^2^_(5 df) _= 37.41, p < 0.001 or X^2^_(1 df) _= 34.65, p < 0.001; KCNQ1, X^2^_(5 df) _= 50.45, p < 0.001 or X^2^_(1 df) _= 49.37, p < 0.001. b) Counts of observed and expected numbers of non-synonymous single nucleotide polymorphisms (nsSNPs). In HERG, fewer nsSNPs occur at completely conserved sites than expected by chance alone (X^2^_(5 df) _= 22.94, p < 0.001 or X^2^_(1 df) _= 10.07, p < 0.05) whereas in KCNQ1, the distribution is not significantly different from the expected count of neutral variation (X^2^_(5 df) _= 1.04, p > 0.05). c) Data were pooled to account for low numbers of expected AAMs at variable sites and significance was confirmed. The distribution of AAMs was significantly different than what would be expected by random chance for both HERG (X^2^_(7 df) _= 26.10, p < 0.001 or X^2^_(1 df) _= 14.17, p < 0.001) and KCNQ1 (X^2^_(7 df) _= 34.74, p < 0.001 or X^2^_(1 df) _= 18.15, p < 0.001).

A greater proportion of AAMs are found at evolutionarily conserved sites, and a smaller proportion are found at variable sites, than would be expected by an underlying neutral process (Figure 3a). Using X^2 ^analysis, the difference between the distributions of observed and expected disease mutations were statistically significant in both channels, even when only the numbers of disease harboring sites were analyzed (ruling out an effect of multiple mutations at highly mutable sites) or when data were pooled to account for low numbers of expected disease mutations in higher variability classes (data not shown). In KCNQ1, nsSNPs distribute randomly but, in HERG, they were significantly underrepresented at completely conserved sites and overabundant at variable positions (Figure 3b).

To ascertain directly whether AAMs associate preferentially with sites that experience low rates of evolutionary change, we utilized an approach similar to the site analysis used above together with codon evolutionary rate obtained from CODEML in PAML [23]. For both channels, AAMs are found at sites with lower evolutionary rates (Figure 3c). In both channels, certain AAMs were located at codons belonging to the 1^st ^and 2^nd ^evolutionary rate categories as well as to variability classes 0, 1 and 2. This implies that, when AAMs are found at variable sites, they may preferentially occur at those with low evolutionary rates.

### Disease mutations are not equally distributed among functional regions of the channels

**Figure 4:**
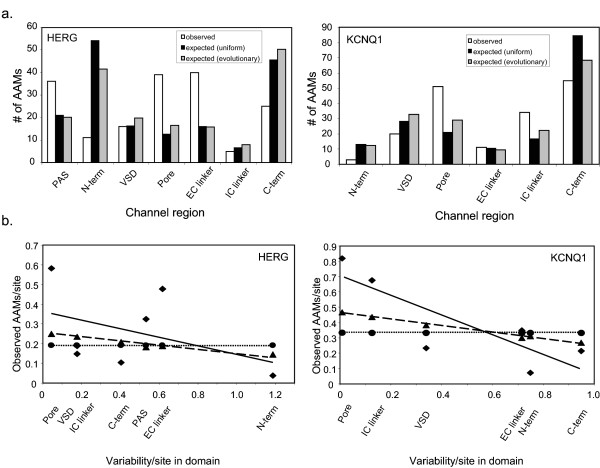
**Arrhythmia-associated mutations are unevenly distributed among functionally conserved regions of HERG and KCNQ1 even after accounting for total length and evolutionary conservation of individual sites therein**. a) Counts of observed number of AAMs per region (white), counts of expected number of AAMS based on a uniform distribution across each gene (black) and expected number of disease mutations based on an evolutionary distribution within each region (gray). Disease mutations are unevenly distributed among different regions of the channel: HERG_uniform _(X^2^_(6 df) _= 145.10, p < 0.001), HERG_evolutionary _(X^2^_(6 df) _= 116.55, p < 0.001), KCNQ1_uniform _(X^2^_(5 df) _= 81.59, p < 0.001) and KCNQ1_evolutionary _(X^2^_(5 df) _= 37.39, p < 0.001). b) Scatter plots showing the relationship between channel region conservation (average variability/site within domain) and the average number of observed disease mutations per site (diamonds) or expected number of disease mutations per site based on a uniform (circles) or evolutionary (triangles) distribution. Dotted and dashed lines indicate fits for expected uniform and evolutionary distribution, respectively. Solid lines represent best-fit regression of observed data. The correlation is significant for KCNQ1 but not for HERG, and the best fit of the KCNQ1 data is significantly different from the other two hypotheses.

Because both channels possess functional regions that are well conserved among voltage-gated channels, we tested for uneven domain distribution of AAMs. KCNQ1 and HERG were divided into six or seven regions, respectively: N-terminus, PAS domain (HERG only), VSD (S1 through S4 transmembrane regions only), pore region (excluding outer turret), extracellular linkers, intracellular linkers and the C-terminus (see Figure 1). Based on the X^2 ^analysis, AAMs in both channels are unevenly distributed among the defined functional domains and, in general, do not support either a uniform pattern (in which the number of randomly occurring mutations are proportional to the total number of residues) or evolutionary pattern (in which the number of randomly occurring mutations are proportional to the total number of conserved residues) (Figure 4a). For both channels, AAMs are overrepresented in the pore region. AAMs are found preferentially in the intracellular (IC) linker of KCNQ1 and the extracellular (EC) linker of HERG, as well as the PAS domain of HERG, but are underrepresented in the N- and C-termini of both channels. This finding is especially striking for KCNQ1 considering 32% of its disease mutations are found in the C-terminus. The number of AAMs in the VSD of HERG and KCNQ1 were not different from the expected number based on a uniform or evolutionary distribution.

To examine whether the overall conservation of a region (average variability/site in domain) exerts a non-additive influence on the disease susceptibility [25], the average number of AAMs per site in a given region of HERG and KCNQ1 was plotted against its average variability per site. These values were correlated for KCNQ1, but not for HERG (Figure 4b). The slope of this relationship in KCNQ1 was greater than those based on the uniform or evolutionary hypothesis. This indicates that AAMs are overabundant in conserved regions and less than expected in variable regions, which suggests that the regional sequence conservation may play a role in KCNQ1 disease susceptibility.

### The chemical severities of AAMs are different than changes observed throughout evolution

**Figure 5:**
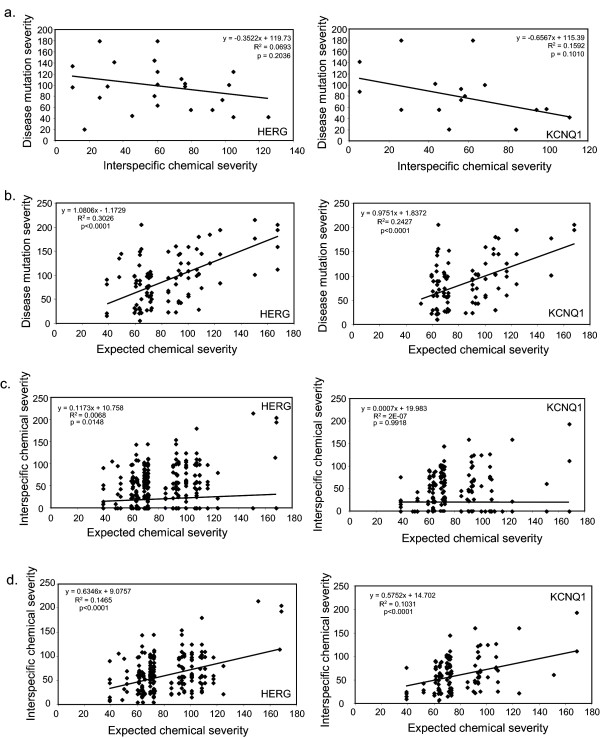
**Arrhythmia-associated mutation and interspecific chemical severities correlate with the expected chemical severity of the reference codon in HERG and KCNQ1, but not with each other**. a) Plot of AAM vs. interspecific chemical severities. No significant correlation is observed for either HERG (p = 0.20) or KCNQ1 (p = 0.10). b) Plots of AAM vs. expected chemical severities. A significant correlation is observed for both HERG (p < 0.0001) and KCNQ1 (p < 0.0001). c) Plots of interspecific vs. expected chemical severities (all sites). A significant correlation is observed for HERG (p = 0.0148) but not for KCNQ1 (p = 0.9918). For both proteins, the correlation (r) statistic, and the slopes of the regression, for interspecific vs. expected chemical severities are significantly smaller (p < 0.001) than those for AAM vs. expected chemical severities ('b', above). d) Plots of interspecific vs. expected chemical severities using only variable sites. A significant correlation is observed for both HERG (p < 0.0001) and KCNQ1 (p < 0.0001). The correlations and slopes of the linear regression are significantly larger (p < 0.001) than those using all sites ('c', above). For both proteins, the slopes of the linear regression are significantly smaller than those for AMM vs. expected chemical severities ('b', above) The correlation (r) is significantly smaller than that for AMM vs. expected chemical severities ('b', above) for HERG (p < 0.05) and not for KCNQ1 (p = 0.058). Significant differences in the average chemical severity were tested using the Mann-Whitney U test whereas the correlation differences between expected chemical severity and disease or interspecific severity were tested for significance using the z-score calculations.

Two sites in each channel are associated with both an AAM and nsSNP. In each case, the AAM and nsSNP involve different amino acid substitutions. Additionally, of the 42 disease sites that overlap with interspecific change, only two sites in HERG and one site in KCNQ1 display an AAM that is the same as an interspecific change, suggesting that the identity of the amino acid contributes to susceptibility to disease. In previous studies, the chemical severity of disease-causing mutations, determined by the Grantham's Scale, were on average larger than interspecific changes, and not correlated with evolutionary changes found at the same sites [21,22]. The average chemical severity of AAMs in HERG and KCNQ1 were also larger than those observed throughout evolution (Table 3). Furthermore, we found no correlation between the chemical severities of both types of change in HERG and KCNQ1 at variable sites that harbor a disease mutation (Figure 5a).

**Table 3:** Average chemical differences of amino acid changes

Gene	Disease	nsSNP	Interspecific
HERG	93.63 (3.99) n = 172	75.50 (10.55) n = 16	67.37 (1.97) * n = 296
KCNQ1	88.29 (3.75) n = 174	52.25 (8.66) † n = 12	58.32 (2.74) * n = 168

*, † indicate a significant difference from disease severity († p < 0.0001, *p < 0.008) as determined by a Mann-Whitney U test. There was no significant difference between the average chemical differences observed in disease vs. polymorphic changes (p = 0.218) for HERG. There was no significant difference between the average chemical differences observed in polymorphic and interspecific amino acid changes in either HERG (p = 0.1441) or KCNQ1 (p = 0.6671).

The chemical severity of amino acid changes may depend on the expected chemical severity of mutations that can arise from a single nucleotide substitution in the codon involved [21]. For KCNQ1 and HERG, the chemical severity of AAMs and expected chemical severity were correlated (Figure 5b). Interspecific and expected chemical severities, however, were not correlated for KCNQ1, and had a very small correlation coefficient for HERG (Figure 5c) (which becomes non-significant when three outliers are removed; not shown). These findings are consistent with previous studies, which suggest that interspecific chemical severity is not influenced by the expected chemical severity of the codon involved but rather by the process of natural selection [21]. Nonetheless, the chemical severities of changes tolerated at variable sites may be influenced by the codon's expected chemical severity. When completely conserved sites were removed (Figure 5d), the expected chemical severity of the involved codon was correlated with interspecific chemical severity, for both HERG and KCNQ1, but to a lesser extent than with disease chemical severity based on slope and correlation co-efficient.

We next compared the average chemical severities of AAMs and nsSNPs (Table 3). We found a significant difference between AAMs and nsSNPs in KCNQ1, but not in HERG, suggesting that other factors, such as location, may play a larger role in causing channel dysfunction for AAMs in HERG compared to KCNQ1. There were no significant differences between nsSNP and interspecific severities in either channel.

### Involvement of specific amino acids in arrhythmogenic disease

**Figure 6:**
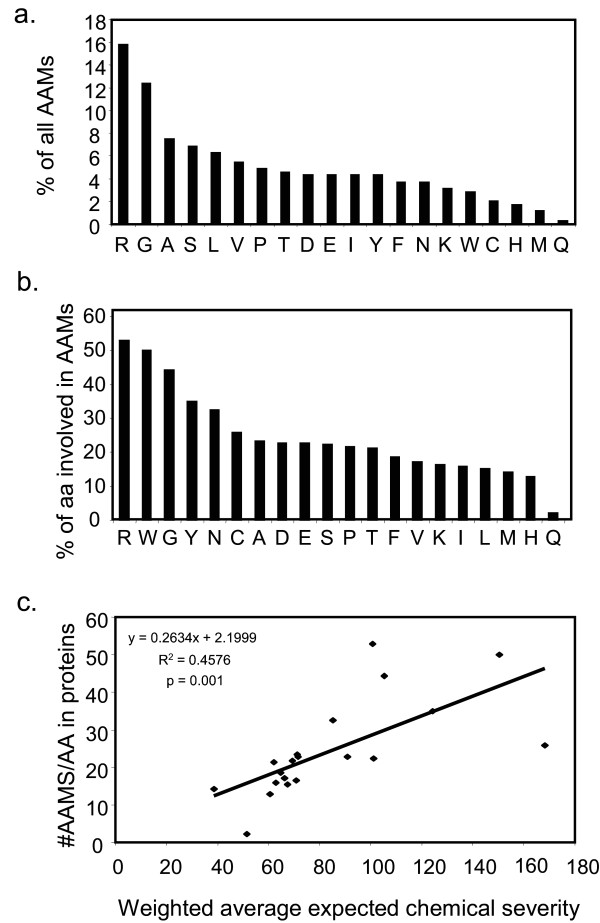
**Mutations occur predominantly at arginine and glycine residues**. a) Percentage of AAMs that occur at a given amino acid residue. b) Relative contribution of a given amino acid residue in HERG and KCNQ1 to AAMs. The total number of AAMs that occur at a given amino acid was divided by the total number of residue sites in the combination of the two protein regions used in the analysis. c) The number of AAMs in KCNQ1 and HERG at each of the twenty residues, proportional to their occurrence in the two channels, is significantly correlated (p = 0.001) with the amino acid's weighted average expected chemical severity.

We next determined which amino acids in HERG and KCNQ1 are targets in arrhythmia-causing mutations. Figure 6 displays the amino acid spectrum of residues involved in disease for both genes. We found that 28% of all AAMs in these two channels have occurred at either a glycine or arginine residue (Figure 6a). This is similar to a broader protein analysis reported previously [37]. The contribution of a particular residue to the overall disease spectrum may be influenced by the proportion of the given amino acid in these proteins. Therefore, we determined the number of disease mutations at a given residue as a proportion of the total number of the residue in both proteins (Figure 6b). Arginine and glycine residues remain highly represented suggesting they are more likely to be involved in a disease phenotype compared to other residues. The proportion of tryptophan residues involved in disease is also high, although the total number of tryptophan residues is low.

The high involvement of some residues in disease may be due to a high mutation rate that occurs at CpG dinucleotides [21] which is a result of a cytosine to thymine transition. This transition is possible in triplets coding for only five of the twenty amino acids, which are: arginine (4/6 codons), serine (1/6 codons), proline (1/4 codons), threonine (1/4 codons) and alanine (1/4 codons). Of the total number of AAMs in HERG and KCNQ1, only 13.3% are due to a C/T transition at a CpG dinucleotide. Of all arginine mutations resulting in disease, 47% are due to this specific nucleotide transition. These numbers suggest that CpG dinucleotides may contribute to the disease process but that this is not the only factor responsible for the high numbers of mutations in this sub-set of amino acids. A role for factors other that CpG dinucleotide hypermutability is also supported by the high involvement of glycines and tryptophans in disease (Figure 6b), which do not possess CpG dinucleotides.

Our findings that the overall chemical severity of AAMs is greater than those of interspecific variation and SNPs, and that the chemical severity of AAMs correlates with expected chemical severity at those individual codons, suggest that chemical severity is on a continuum and that a threshold severity exists which, once crossed, results in disease. We might then expect that whether mutations at specific amino acids cause disease at all also depends on the expected chemical severity of the involved codon. To examine this, the proportion of total AAMs at a particular residue was plotted against the calculated weighted average of expected chemical severity (Figure 6c). A significant, positive correlation was found between these suggesting that expected chemical severity of a site contributes to the probability of obtaining a disease mutation, as well as to the severity of that mutation.

## Discussion

In this study, we show that that AAMs are overabundant at evolutionarily conserved and slowly evolving sites, which are likely critical for channel function and thus intolerant of changes in amino acid sequence. Because KCNQ1 and HERG are highly conserved, an underlying neutral mutational process could produce large numbers of mutations at conserved sites. Our data provide the quantitative backing to support an over representation of AAMs at evolutionarily conserved positions in these channels. A smaller than expected, but still substantial, numbers of AAMs were found at sites that show interspecific variation. However, only two sites in HERG and one in KCNQ1 are converted to residues that are both an AAM and an interspecific change. Thus, the identity of the residue, rather than its location, is most responsible for producing the disease at sites that have undergone evolutionary change.

HERG and KCNQ1 possess structurally defined domains with specific functions that have been strongly preserved throughout the course of evolution [36,38]. We found that AAMs are found preferentially in some domains, even after accounting for their size and evolutionary conservation. In both channels, the pore region possesses an overabundance of mutations, while both N- and C-termini possess an underabundance of mutations. The extracellular linker region had an overabundance of mutations in HERG, whereas the intracellular linker had an overabundance of mutations in KCNQ1. This implies that the extracellular linker may be more important to overall function in HERG, whereas the intracellular linker is more critical for function in KCNQ1. The overabundance of mutations in the PAS domain, not present in KCNQ1, highlights its functional importance and suggests that that the addition of a functionally important domain in a protein can increase susceptibility to disease. Overall regional conservation, which takes into account the average variability per site in a domain, contributes to the uneven regional distribution of disease-causing mutations in some proteins [25], but we found this only for KCNQ1 (Figure 4b). Therefore, factors other than domain size, site conservation and regional conservation must influence the domain specific distribution of AAMs in HERG, and possibly in KCNQ1 as well.

NsSNPs are underabundant at conserved sites in HERG, but distribute randomly in KCNQ1. The latter finding may be due to a small sample size, although the numbers of nsSNPs analyzed in HERG were similarly small. This difference between the channels may be because nsSNPs are less disruptive when located at conserved sites in KCNQ1, or because conserved sites in HERG are less tolerant to amino acid changes. Nevertheless, the distribution of analyzed nsSNPs in both channels suggests that they are phenotypically neutral. The different patterns of nsSNP distribution between the KCNQ1 and HERG underscore the need to identify and quantitatively analyze the distribution of more nsSNPs on each protein, and to ascertain their impact on channel function and arrhythmia susceptibility. In HERG, it is known that some, but not all, polymorphisms may alter channel function [39], and also contribute to an increased QT interval duration [40].

In both HERG and KCNQ1, AAMs are chemically more severe than interspecific changes tolerated throughout evolution or polymorphisms that are not associated with disease. Codons with a higher expected chemical severity are associated with disease mutations with a high chemical severity. These data are in keeping with those found previously in rhodopsin [21] and suggest that the intrinsic potential of the involved codon contributes to disease chemical severity.

We also provide novel evidence that the expected chemical severity of a codon contributes to the overrepresentation of certain amino acids in HERG and KCNQ1 AAMs, especially arginine, tryptophan and glycine. In a recent study of 437 proteins, these three amino acids were also highly overrepresented in DAMs [37]. Therefore, we predict that expected chemical severity plays an important role in determining the propensity of a given codon to cause disease in many proteins, in addition to other factors such as the residue's roles in biosynthesis, function and stability of the channels. Finally, the chemical severities of interspecific changes in KCNQ1 and HERG also correlate with those expected for the codons, when only evolutionarily variable sites are considered. These novel data argue that, in addition to a predicted role of natural selection, the expected chemical severity of the codon contributes to variation observed over the course of evolution in these channels. Despite the presumed predominance of natural selection, the genetic code has been shown to influence the mutational process when evolutionary divergence is low [41]. These data are significant given the uncertainty as to the role of natural selection versus non-adaptive forces in shaping genotypic and phenotypic variation [42,43].

Our analyses may be influenced by the fact that some mutations may, in a systematic way, never be detected. For example, AAMs that lead to death before natural birth, or to SUDs, may never be identified unless the fetus or victim is subsequently screened for arrhythmogenic mutations. This could, in turn, reduce the number of observed mutations unique to certain sites or functional domains. The evaluation of AAMs has broadened, and identification of mutations in KCNQ1 and HERG, and in other candidate genes, associated with SIDs and SUDs has been carried out [14-17]. Identification of AAMs in a more broad population may reveal a different sub-set of mutations that localize to unique regions within the channels, or more strongly support the susceptibility of sites and functional domains identified in this study to arrhythmogenic mutations.

## Conclusion

Our study represents the first quantitative evolutionary and chemical severity analysis of AAMs in the HERG and KCNQ1 potassium channel genes. Unlike nsSNPs, AAMs preferentially locate to evolutionarily conserved, and functionally important, sites and regions within HERG and KCNQ1, and are chemically more severe than changes which occur in evolution. Expected chemical severity may contribute to the overrepresentation of certain residues in AAMs, as well as to changes observed throughout evolution.

Our findings, together with those from other studies, suggest that novel DAMs and AAMs may be recognized quickly by surveying naturally occurring variation among species [21]. If a SNP identified in an individual does not appear in other species at that position, then it is likely to be disease-causing. The location of AAMs (to conserved or variable regions and/or residues) may correlate with clinical severity or other characteristics of the diseases. In the case of Long QT syndrome, genotype and specific mutations have been shown to contribute to phenotype [44-47] and the underlying genetic defects contribute to risk stratification, prevention and therapy [12,48]. Unfortunately, there is still considerable variation in Long QT phenotype and age of disease onset [13]. Therefore, continued discovery and mapping of mutations, as done in this study, along with parallel studies on disease phenotype will ultimately lead to a better understanding of the genotype-phenotype relationship, help to better predict the outcome of novel disease mutations and aid in development of mutation-specific therapies.

## Authors' contributions

Together, HJ and EA designed the study, interpreted the data and drafted the manuscript. HJ performed the data collection and the evolutionary and statistical analyses. Both authors have read and have approved the final manuscript.

## Supplementary Material

Additional file 1 **A list of AAMs and nsSNPs in KCNQ1 and HERG used in the analyses**. A list of all disease mutations and SNPs which were used in these analyses along with their location within each channel, the disease phenotype with which they are associated and the database reference from which they were collected. Click here for file
